# BiDaS: a web-based Monte Carlo BioData Simulator based on sequence/feature characteristics

**DOI:** 10.1093/nar/gkt420

**Published:** 2013-05-28

**Authors:** Maria D. Paraskevopoulou, Ioannis S. Vlachos, Emmanouil Athanasiadis, George Spyrou

**Affiliations:** ^1^Biomedical Informatics Unit, Biomedical Research Foundation, Academy of Athens, 4 Soranou Ephessiou, 115 27 Athens, Greece, ^2^Department of Computer and Communication Engineering, School of Engineering, University of Thessaly, 382 21 Volos, Greece and ^3^Laboratory for Experimental Surgery and Surgical Research “N.S. Christeas”, Medical School of Athens, University of Athens, 115 27 Athens, Greece

## Abstract

BiDaS is a web-application that can generate massive Monte Carlo simulated sequence or numerical feature data sets (e.g. dinucleotide content, composition, transition, distribution properties) based on small user-provided data sets. BiDaS server enables users to analyze their data and generate large amounts of: (i) Simulated DNA/RNA and aminoacid (AA) sequences following practically identical sequence and/or extracted feature distributions with the original data. (ii) Simulated numerical features, presenting identical distributions, while preserving the exact 2D or 3D between-feature correlations observed in the original data sets. The server can project the provided sequences to multidimensional feature spaces based on: (i) 38 DNA/RNA features describing conformational and physicochemical nucleotide sequence features from the B-DNA-VIDEO database, (ii) 122 DNA/RNA features based on conformational and thermodynamic dinucleotide properties from the DiProDB database and (iii) Pseudo-aminoacid composition of the initial sequences. To the best of our knowledge, this is the first available web-server that allows users to generate vast numbers of biological data sets with realistic characteristics, while keeping between-feature associations. These data sets can be used for a wide variety of current biological problems, such as the in-depth study of gene, transcript, peptide and protein groups/families; the creation of large data sets from just a few available members and the strengthening of machine learning classifiers. All simulations use advanced Monte Carlo sampling techniques. The BiDaS web-application is available at http://bioserver-3.bioacademy.gr/Bioserver/BiDaS/.

## INTRODUCTION

The advent of new and powerful workstations and supercomputers has enabled systems biologists, computational biologists and bioinformaticians to design and implement complex biological models (1). Nevertheless, the efficiency of these models requires significant amounts of biological data/features, to simulate accurately biological functions and validate the derived outcomes. Quality and quantity of data are most of the times equally important during the design and implementation of computational models and can affect significantly the validation process, especially in cases where high dimensional data are required. Furthermore, biological features exhibit frequent intrinsic associations, with a high degree of at least two-dimensional (2D) and three-dimensional (3D) correlations.

In recent years, several sequence (AA/DNA/RNA)-generating web servers have been developed. Namely, in the *RandSeq* web server (2), users are able to generate random AA sequences. Nevertheless, sequence composition parameters are set either manually or from one specific protein. Furthermore, the server interface provides single sequences, rendering it improper for use in cases where large amounts of sequences are required. On the other hand, FaBox server (3), allows researchers to generate any number of random DNA sequences. However, fractions of nucleic acids have to be manually selected, while RNA/AA sequences are not supported. In addition, sequence lengths are randomly chosen without following a given distribution. CorGen (4) is a webserver application that generates sequences based on GC-content correlations. The user-defined input data are restricted to a single DNA sequence, while shorter sequences cannot be examined, as the model performs sequence analysis according to long-range correlations. In addition, several stand-alone applications have been developed such as GenRGenS (5) and rMotifGen (6) that generate sequences based on Monte Carlo simulation theory. However, no web service is available for these implementations, and the user has to download and install them locally.

BiDaS webserver represents a next generation of webservers, incorporating advanced simulation methods, such as hidden Markov models (HMMs) and Monte Carlo (MC) techniques. The implementation of a state-of-the-art simulation pipeline can be a resource- and time-demanding task. BiDaS simulator provides a user-friendly interface helping users to accurately generate data that follow evident, as well as hidden properties of the original data sets. Furthermore, the implemented 2D and 3D Monte Carlo Rejection techniques and methodologies, such as Cholesky decomposition, can provide an adapted framework for data generation that can keep high order correlations potentially present in the initial data.

## BiDaS WEB SERVER

The BiDaS web application is divided into three main sections: (i) *De novo* simulation of Sequences, (ii) *De novo* simulation of Numerical Features and (iii) Sequence Driven simulation of Numerical Features. In addition, BiDaS offers a personalized user workspace, where uploaded and generated data are stored. The application front-end was implemented using *html* and *PHP*, whereas all computations and simulations have been developed in R (http://www.r-project.org/).

‘*De novo* simulation of Sequences’: in this section, researchers are able to upload a file to the workspace that contains either DNA/RNA, or AA sequences in text or FASTA format. Following the file upload, the users can analyze the provided sequences and generate MC-simulated sequences having identical characteristics with the original samples, such as length distribution and nucleotide/AA compositional probabilities. To this end, the server analyzes and simulates original distributions using functions belonging to the logspline family. These distributions are subsequently sampled, to generate characteristics for each simulated sequence. The resulting MC-simulated sequences are created by using each feature instance (e.g. length and nucleotide/AA probability) as a blueprint. Moreover, this section enables the user to realize de novo sequence generation based on Hidden Markov models. The input sequences are initially aligned with the Clustal Omega algorithm (7), to allow the construction of an HMM profile. Subsequently, random sequences are generated using the HMM profile, produced in the previous step from the *HMMER* package (8,9). This web server option is specifically designed for simulation of homologous proteins or DNA/RNA/AA sequences presenting common motifs and/or sequence similarities.

‘*De novo* simulation of Numerical Features’: researchers in numerous settings directly use numerical (e.g. extracted numerical features) and not sequence data. In this section, users can directly upload numerical data sets, such as amino acid composition, hydrophobicity, polarizability, charge, Van der Waals volume, polarity, composition, transition and distribution properties, GC content or any other feature and sequence property. The web server can analyze and simulate data following identical feature distributions with the original data set (1D simulation), while safeguarding all 2D and 3D between-feature correlations observed in the provided data. Correlated features are generated using Cholesky decomposition. The web server in this section, as in all available modules, can help users analyze the original and simulated data, identify correlated features, detect normally distributed characteristics (1D, 2D and 3D multivariate normal distributions), and visualize the original data sets as well as all MC-simulated data. User uploaded data sets and generated data are stored into the personalized user workspace.

‘Sequence Driven simulation of Numerical Features’: In this section, users having DNA/RNA and AA sequences can calculate up to ten 10 numerical features using pseudo-AA composition (10,11), 38 DNA/RNA features based on conformational and physicochemical DNA features from B-DNA-VIDEO database (12) and 122 DNA/RNA features based on conformational and thermodynamic dinucleotide properties from DiProDB database (13). These features are concurrently calculated for sequences belonging to the original user-provided data set, as well as for any sequences generated using the ‘De novo simulation of Sequences’ module. To this end, BiDaS implements an MC rejection method comprising quintile 1D outlier detection and multidimensional Mahalanobis distance-based rejection in cases of 2D correlated features. BiDaS identifies and rejects randomly sampled sequences, not following feature distributions and multi-dimensional feature associations observed in the original data set. These MC-simulated sequences can follow the original length and nucleotide/AA compositional distributions, any given combination of physicochemical, thermodynamic and conformational properties and their in-between correlations. The server provides the option to work subsequently with the derived simulated sequences, their numerical features or both.

## PERFORMANCE STUDIES

BiDaS can be used in various bioinformatics and computational biology studies in biomolecule level (e.g. RNA transcripts, or peptide subclasses) or in a systems biology setting. Whenever the number of available samples is smaller than what is required to derive biologically meaningful results, to perform complex simulations, to formulate systems biology models or to implement classifiers, BiDaS is able to use the initially available group of gene, transcript and peptide sequences or any feature characteristics, and simulate vast numbers of samples with identical properties.

For instance, a researcher is studying an experimentally identified small set of non-coding transcripts, presenting an enhancer-like function for protein-coding genes. However, this data set can be substantially smaller than what is required to train, test and validate a machine learning classifier, able to detect this transcript subclass. In this case, BiDaS can be used to derive an arbitrarily larger set of RNA molecules following similar composition, conformational features and any other user-defined properties, enabling the implementation of the transcript classifier model.

In a practical application scenario, BiDaS performance has been evaluated in real numerical and sequence data sets. In a first case study, the web server was used to generate *MC AA* sequences. Subsequently, MC-simulated sequences were differentiated as antimicrobial or non-antimicrobial, using specifically implemented machine learning classifiers. More precisely, two sets, comprising 1464 proteins annotated as antimicrobial and 3888 proteins annotated as non–anti-microbial, were collected from SwissProt database (14). The former set was further divided into a training set and a test set containing 984 (SwissP-*Anti-Train*) and 480 (SwissP*-Anti-Test*) sequences, respectively. The latter set was separated into two subsets, training and test each including 2000 (SwissP-NON-Anti*-Train*) and 1888 (SwissP-NON-Anti*-Test*) sequences, respectively. Fourth degree (4^th^) pseudo-AA composition features were calculated for all sets using the BiDaS online interface (Sequence Driven simulation of Numerical Features’ module). Subsequently, three classification algorithms (Bayesian and a Probabilistic Neural Network (*PNN*) with Gaussian and Exponential kernel) were implemented in R. All algorithms were trained using the same training data set (SwissP-*Anti-Train*) and validated against the SwissP*-Anti-Test* data set. For each classification scheme, Accuracy, F-Score and Matthews Correlation Coefficient (*MCC)* were estimated for all possible combinations of the selected four features.

**Figure 1.**
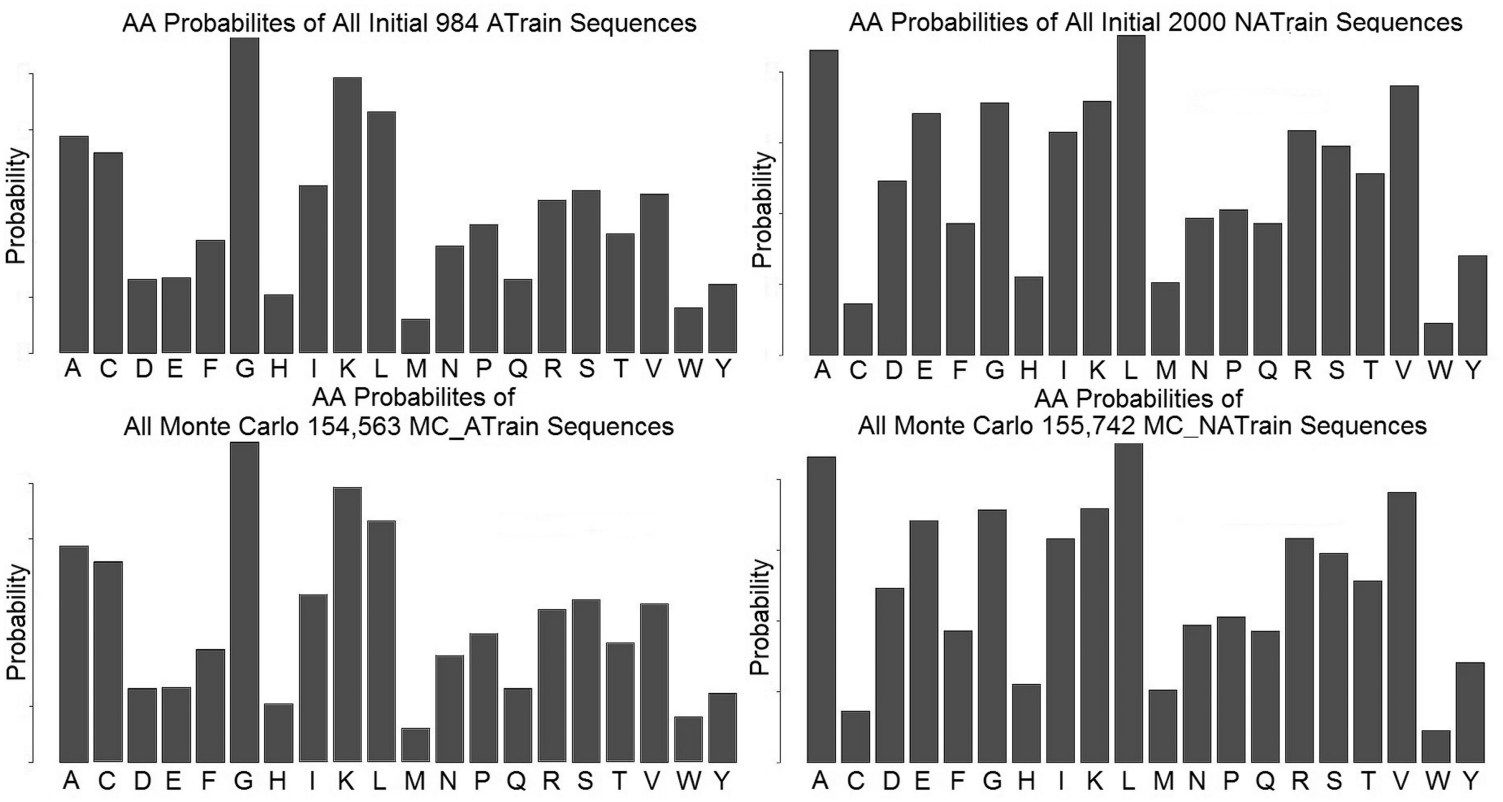
Histograms of AAs within the initial and the MC-generated training data sets.

BiDaS web server was used to generate 400 000 antimicrobial and non-antimicrobial sequences (200 000 for each data set) following length distributions and AA compositional probabilities of the initial SwissP-*Anti-Train* and SwissP-NON-Anti*-Train* training data sets. Subsequently, 4^th^ degree pseudo-AA composition were calculated. BiDaS MC-rejection method was used, rejecting all sequences not having identical pseudo-AA distributions and inter-feature correlations with the original data. Specifically, 154 563 MC-simulated anti-microbial (*MC-Anti-Train*) and 155 742 MC-simulated non-antimicrobial sequences (*MCNON-Anti-Train*) were finally generated that conformed to the selected criteria. Histograms of AAs within the initial and the MC-generated training data sets are presented in Figure 1.

The resulting features, derived from the positive (antimicrobial) and negative (non-antimicrobial) MC-simulated sequences, were used to train again a Bayesian and the two PNN classifiers (MC-Anti-Train and MC-NON-AntiTrain). The classifiers were trained using solely MC-generated features, without the inclusion of any of the original SwissProt-derived anti-microbial sequences. The performance of the classification algorithms was validated on the same test sets (SwissP-Anti-Test & SwissP-NON-Anti-Test) and with the same metrics used in the previous experiment. It is noteworthy that the performance of the classifiers being trained on real or simulated data was comparable. Detailed results are presented on Table 1.

**Table 1.** Classification results of best feature combination for each of the three classifiers using real as well as simulated sequences in the training process

Classifier	Best feature combination	Training data set	Accuracy	F-Score	*MCC*
Bayesian	PseudoAA1-AA2	Real	75.63	88.67	0.35
		Monte Carlo	75.04	88.72	0.35
PNN with Gaussian Kernel	PseudoAA1-AA2	Real	78.76	88.45	0.39
		Monte Carlo	77.03	87.42	0.34
PNN with Exponential Kernel	PseudoAA1-AA2	Real	80.95	88.20	0.41
		Monte Carlo	78.42	85.92	0.32

**Figure 2.**
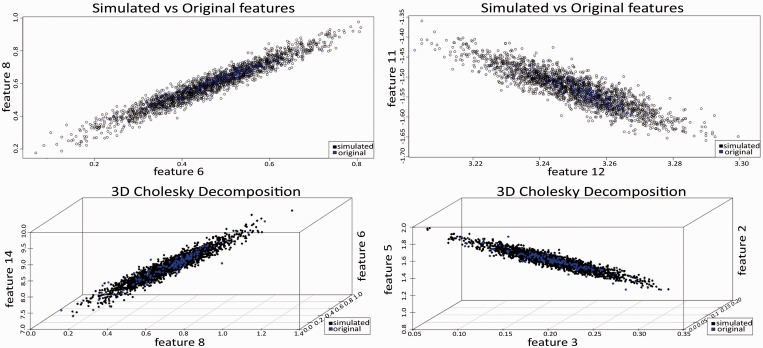
Simulated 2D and 3D correlated features using Cholesky Decomposition. Original features are represented in all diagrams with blue points, while MC-simulated values are depicted in black. In-between correlations are preserved in the simulated feature sets.

In a second case study, BiDaS web server was used to simulate properties/features for a set of 153 3′untranslated regions (3′UTRs) of ribosomal protein-coding transcripts. The computations were performed on the transcript variants with the longest 3′UTR sequence. BiDaS has generated MC-simulated features based on an initial set of 22 conformational and thermodynamic dinucleotide properties derived from DiProDB database. In this case study, ‘*De novo* simulation of Numerical Features’ has been selected. The final set of the simulated data was composed of 4000 instances; half of them were generated with Cholesky Decomposition based on 2D and the rest on 3D correlations of the original features. Graphical representations of the simulated 2D, 3D correlated versus the original data are presented in Figure 2. Additionally, Spearman's rho coefficient values were estimated to validate the performance of the simulations. In-between correlations of the original features were also preserved in the simulated data set.

## CONCLUSION

BiDaS web server is designed to support Monte Carlo AA or DNA/RNA sequence generation, as well as numerical feature simulations. To our knowledge, there are no existing web applications with that extent of functionalities, as the currently available tools restrict their simulations to either sequence or feature-specific properties. We strongly believe that BiDaS will empower researchers with state-of-the-art sequence- and feature-generation tools, as well as simulation methodologies without the necessity to develop and maintain advanced computational pipelines. BiDaS will be a major tool when building complex biological models or developing computational intelligence schemes especially in cases with small or rare experimental/clinical data sets.

## FUNDING

Funding for open access charge: Biomedical Research Foundation of the Academy of Athens. Emmanouil Athanasiadis and George Spyrou are supported by the NSRF 2007–2013, co-funded by the European Regional Development Fund and national resources, under grant “Cooperation” [No. 09ΣYN-11-675].

*Conflict of interest statement.* None declared.
